# The international, prospective CytOSorb^Ⓡ^ treatMent Of critically ill patientS (COSMOS) registry: Interim results from the first 150 patients

**DOI:** 10.1016/j.jointm.2025.05.001

**Published:** 2025-06-27

**Authors:** Ricard Ferrer, Matthias Thielmann, Andreas Kribben, Moritz Unglaube, Bartosz Tyczynski, Julian Kreutz, Andreas Baumann, Ulf Guenther, Dietrich Henzler, Thomas Kirschning, Aschraf El-Essawi, Thomas Günther, Martin Bellgardt, Gabriella Bottari, Filippo Aucella, Jorge Hidalgo, Jean-Louis Teboul, Dana Tomescu, Teresa Klaus, Weihong Fan, Jörg Scheier, Efthymios N. Deliargyris, Fabio Silvio Taccone

**Affiliations:** 1 Intensive Care Department, Vall d'Hebron University Hospital, Shock, Organ Dysfunction and Resuscitation Research Group (SODIR), Barcelona, Spain; 2 Department of Thoracic and Cardiovascular Surgery, West German Heart & Vascular Center Essen, University Hospital Duisburg-Essen, Essen, Germany; 3 Department of Nephrology, University Duisburg-Essen, University Hospital Essen, Essen, Germany; 4 Department of Intensive Care, Helios Dr. Horst- Schmidt Hospital Wiesbaden, Wiesbaden, Germany; 5 Department of Medical Intensive Care, University Hospital Essen, Essen, Germany; 6 Department of Cardiology, Angiology and Intensive Care Medicine, Philipps University of Marburg, University Hospital, Marburg, Germany; 7 Department of Anesthesiology, Intensive Care Medicine and Pain Management, BG University Hospital Bergmannsheil, Medical Faculty of Ruhr University Bochum, Bochum, Germany; 8 University Hospital of Anesthesiology and Intensive Care, Hospital Oldenburg, Oldenburg, Germany; 9 Department of Anesthesiology, Surgical Intensive Care, Emergency and Pain Medicine, Ruhr-University Bochum, Hospital Herford, Herford, Germany; 10 Department of Cardiothoracic Surgery, Heart and Diabetes Center NRW, Bad Oeynhausen, Germany; 11 Department of Thoracic and Cardiovascular Surgery, University Medical Center Goettingen, Goettingen, Germany; 12 Department of Cardiovascular Surgery, German Heart Center Munich, School of Medicine & Health, TUM University Hospital, Technical University of Munich, Munich, Germany; 13 Department of Anesthesiology and Intensive Care Medicine, St Josef-Hospital Bochum, University Hospital of the Ruhr-University Bochum, Bochum, Germany; 14 Pediatric Intensive Care Unit, Children Hospital Bambino Gesù, IRCCS, Rome, Italy; 15 Department of Nephrology and Dialysis, “Casa Sollievo della Sofferenza” Foundation, Scientific Institute for Research and Health Care, San Giovanni Rotondo, Italy; 16 General Intensive Care Unit and COVID-19 Unit, Belize Healthcare Partners, Belize City, Belize; 17 Paris-Saclay Medical School, Paris-Saclay University, Le Kremlin-Bicêtre, France; 18 Department of Anesthesiology and Critical Care III, “Carol Davila” University of Medicine and Pharmacy, Fundeni Clinical Institute, Bucharest, Romania; 19 Medical Affairs, CytoSorbents Europe GmbH, Berlin, Germany; 20 Medical Affairs, CytoSorbents Corporation and CytoSorbents Medical Inc., Princeton, USA; 21 Department of Intensive Care, Hôpital Universitaire de Bruxelles (HUB), Université Libre de Bruxelles (ULB), Brussels, Belgium

**Keywords:** Cytosorb, Hemoadsorption, Hemoperfusion, Adsorption, Blood purification, Hyperinflammation

## Abstract

**Background:**

The CytOSorb^Ⓡ^ treatMent Of critically ill patientS (COSMOS) registry is an observational, prospective, multicenter, international real-world data collection platform executed in countries where the CytoSorb^Ⓡ^ (CS) 300 mL device is approved and routinely used in everyday clinical practice. This study aims to investigate utilization patterns of the hemoadsorption device and associated outcomes in critical care.

**Methods:**

Since July 2022, patients who were treated with CS as part of their intensive care treatment were enrolled from 16 sites in Germany, Italy, and Spain in the registry. After informed consent, real-world clinical data are systematically collected at multiple intervals, including 24 h before CS start, during CS treatment, and 24 h post-CS treatment, as well as at discharge from intensive care unit (ICU) and hospital discharge, and final follow-up on day 90. Vital status was assessed as ICU survival, 30-day survival, overall hospital survival, and 90-day survival. We compared details on the type of extracorporeal circuit used, device flow rate, anticoagulation regimen, vasopressor requirements, fluid balance, ratio of partial pressure of oxygen in arterial blood to the fraction of inspiratory oxygen concentration (P/F ratio), myoglobin in the rhabdomyolysis cohort and bilirubin in the liver failure cohort before and after CS treatment. Safety of the device was assessed by investigator-reported device-related adverse effects. Data were presented as either mean ± standard deviation or as median with interquartile range (IQR).

**Results:**

A total of 150 patients were enrolled, 23 patients did not have any data entry by the time of the data readout for this interim analysis conducted and therefore had to be excluded from the analysis (33 % female, mean age [59±17] years). CS indications included septic shock (57.6 %), cardiogenic shock (12.9 %), and rhabdomyolysis (10.6 %). CS was mainly integrated with renal replacement therapy (82.8 %). Median Acute Physiology and Chronic Health Evaluation II score was 23 (IQR: 17–29), Sequential Organ Failure Assessment score 12 (IQR: 9–15), and ICU stay 20 (IQR: 11–33) days. Median interleukin-6 decreased significantly from 862.4 (IQR: 142–97,457) pg/mL in the 24 h before CS treatment to 202.8 (IQR: 42–3247) pg/mL in the 24 h post-CS treatment (*P* <0.0001). Post-CS, lactate and creatinine levels significantly decreased, fluid balance improved from 1386 (IQR: 220–3168) mL to 275 (IQR: -768–1846) mL (*P* <0.0001) and median P/F ratio increased from 132 (IQR: 68–208) mmHg to 189 (IQR: 115–260) mmHg (*P* <0.0001). Norepinephrine requirements reduced from 0.30 (IQR: 0.18–0.46) µg/(kg·min) to 0.19 (IQR: 0.10–0.33) µg/(kg·min) (*P*=0.0003). In rhabdomyolysis patients, myoglobin decreased from 18,976 (IQR: 1934–34,275) to 835 (IQR: 623–5925) µg/L (*P*=0.0273). Observed ICU mortality was 35 %, lower than predicted by baseline scores.

**Conclusions:**

The COSMOS registry highlights CS-associated improvements in lactate, creatinine, norepinephrine needs, fluid balance, and oxygenation. Mortality was favorable compared with risk-based predictions.

Trial registration Clinicaltrials.gov Identifier: NCT05146336

## Introduction

In critical care settings, prolonged and excessive inflammation, driven by a persistent release of various inflammatory mediators, is a major factor leading to multiple organ failure and increased mortality risk.^[^^1^^,^^2^^]^ Blood purification techniques, including hemofiltration, hemoadsorption, and plasma-processing methods, such as plasmapheresis and plasma exchange, have emerged as therapeutic strategies to manage such inflammation.^[^^3^^]^ These methods aim to remove inflammatory mediators from the bloodstream, thereby reducing their detrimental effects on organs and restoring immune balance.^[^^4^^,^^5^^]^ Hemoadsorption, specifically, involves removing circulating inflammatory molecules such as cytokines on the surface of a sorbent material. Different hemoadsorption methods vary in their ability to retain specific molecules, the need for supplementation of physiological blood components, and their technical and cost implications.^[^^6^^]^

One such device, the CytoSorb^Ⓡ^ (CS) hemoadsorber, is a Conformité Européenne (CE)-marked whole blood hemoadsorption system that has seen widespread use since its introduction in 2011. To date, over 250,000 devices have been used in clinical practice without any safety concerns identified in post-market surveillance.^[^^7^^]^ A recent randomized controlled study in healthy volunteers provided definitive mechanistic evidence that CS can decrease systemic cytokine levels following intravenous administration of endotoxin in an established sepsis model.^[^^8^^]^ In addition, CS has been approved and is currently used for the removal of bilirubin, myoglobin, and certain antithrombotic drugs during cardiothoracic surgeries involving cardiopulmonary bypass.^[^[9], [10], [11]^]^ The CS device can be integrated into various extracorporeal circuits, including continuous renal replacement therapy (CRRT), dedicated hemoperfusion circuits, extracorporeal membrane oxygenation (ECMO) circuits, and cardiopulmonary bypass circuits, making it a versatile tool in critical care.

Although hemoadsorption therapies are increasingly used worldwide, unanswered crucial questions remain about optimal timing, dosing, and specific patient populations who are likely to respond to the therapy with reproducible clinical benefits.^[^^12^^]^ Due to the heterogeneity of treatment effects in critically ill patients, registries such as the international CytOSorb treatMent Of critically ill patientS (COSMOS) are invaluable for complementing much-needed data from controlled clinical trials as they provide real-world evidence (RWE), by reflecting routine clinical practice.^[^^13^^,^^14^^]^ In addition, RWE collection is essential for post-market surveillance as required by Medical Device Regulations.^[^^15^^]^

Since July 2022, the COSMOS registry has been prospectively enrolling consecutive critically ill patients, including children, who have undergone hemoadsorption treatment as part of their standard care. This interim analysis presents data from the first 150 patients enrolled in the registry, providing insights into real-world use of the device and associated clinical performance.

## Methods

### Registry study design

The COSMOS registry is an observational, prospective, multicenter, international data collection platform executed in countries where the CS 300 mL device is approved and routinely used in everyday clinical practice. It aims to collect real-world clinical data on the use of the device, capturing treatment patterns, outcomes, and safety to enhance knowledge, improve patient care, and facilitate future research, including randomized controlled trials. It started with the inclusion of the first patient on July 15, 2022 and is now actively enrolling in four countries (Germany, Spain, Italy, and Portugal). Sites were selected based on CS use as part of their routine everyday practice, expected patient numbers to be enrolled, study conduct expertise, electronic data collection capabilities, and interest in participation. The use of CS is per standard practice and not directed by the registry protocol. Training was provided to all site personnel, including principal investigators, sub-investigators, and clinical research coordinators, to ensure device use according to the best current practice.^[^^7^^,^^15^^]^ Investigators are also encouraged to review the most recent CS Instructions for Use to assess contraindications and precautions. To avoid selection bias and represent real-world practice, sites are asked to include consecutive patients without preselection. All clinical data from patients treated after Institutional Review Board/Ethics Committee (IRB/IEC) approval are considered prospective data. Informed consent was obtained from all patients involved in the study.

Inclusion criteria: (1) planned or actual CS 300 mL device utilization; (2) informed consent for prospective registry participation. Exclusion Criteria: (1) use of the CS 300 mL device for antithrombotic removal only; (2) intraoperative use of CS 300 mL device during cardiac surgery only; (3) the occurrence of a complication or other medically justified circumstance that arises after written informed consent has been obtained from the patient and before or during the planned therapy and as a result of which the use of CS adsorber is contraindicated or no longer appropriate.

The registry collects patient-level data with CS use in various clinical indications with more than one indication possible in some patients (Table 1).

**Table 1:** Different indications included in the COSMOS registry

Category	Clinical indications
Hyperinflammation	Septic shock Post-operative vasoplegic shock Hemophagocytic lymphohistiocytosis Cytokine release syndrome Chimeric antigen receptor therapy CRS
Cardio/pulmonary	Cardiogenic shock ARDS ECMO/ECLS
Liver/kidney	ALF/ACLF Rhabdomyolysis
Infectious disease	COVID-19 Influenza Dengue
Others	Burns Pancreatitis Overdose drug removal

ARDS: Acute respiratory distress syndrome; ALF: Acute Liver Failure; ALCF: Acute-On-Chronic Liver Failure; CRS: Cytokine release syndrome; COSMOS: CytOSorb^Ⓡ^ treatMent Of critically ill patientS; COVID-19: Coronavirus infectious disease 2019; ECLS: Extracorporeal life support; ECMO: Extracorporeal membrane oxygenation.

### Study outcomes and data collection

Data are systematically collected at multiple intervals, including 24 h before CS start, during CS treatment, and 24 h post-CS treatment, as well as in the intensive care unit (ICU) and at hospital discharge, and final follow-up at day 90.

Vital status is assessed as ICU survival, 30-day survival, overall hospital survival, and 90-day survival.

Additional outcomes assessed in this interim analysis include: details on the type of extracorporeal circuit used, device flow rate, anticoagulation regimen, details on vasopressor requirements, change in fluid balance, change in ratio of partial pressure of oxygen in arterial blood to the fraction of inspiratory oxygen concentration (P/F ratio), change in myoglobin in rhabdomyolysis cohort, change in bilirubin in liver failure cohort, and safety of the device assessed by investigator-reported device-related adverse effects until ICU discharge or death, whatever comes first.

### Statistical analysis

A pre-specified statistical analysis plan was followed to summarize and analyze the data variables. Since the registry covers many critical care settings (even rare settings), no *a priori* power calculation was performed. As this is an ongoing study there were missing values for different parameters at the time of data extraction and the actual available number of data was used as the denominator for any percentage calculation. For continuous variables, median with interquartile range (IQR) or mean with standard deviation (SD) were tabulated. For categorical variables, the counts and proportions of each value were tabulated. Baseline was defined as time of ICU admission for demographics, medical history, comorbidities, and calculation of standardized risk scores. Pre-CS therapy period was defined as the 24 h before the CS start for vasopressor and fluid balance, and the latest assessment for lab values and blood gas. Post-CS therapy is defined as the 24 h after the end of CS therapy for vasopressor and fluid balance, and the earliest assessment for lab values and blood gas. Absolute change from pre-CS therapy was calculated as the post-CS measurement minus the pre-CS value. The Wilcoxon signed-rank test was used to compare paired data (pre- and post-CS therapy) to assess changes associated with CS treatment. All *P*-values are considered as *post hoc* and exploratory to gain a deeper understanding of the clinical benefit of CS use and not adjusted to account for the increased risk of Type I errors. *P*-values were rounded and displayed using four decimals. If a *P*-value <0.0001 occurred, it would be shown in tables as <0.0001. All analyses were performed using SAS 9.4 (SAS, Cary, NC).

## Results

### Patient characteristics

A total of 150 patients were enrolled at 16 sites in Germany, Spain, and Italy. However, 23 patients did not have any data entry by the time of the data readout for this interim analysis conducted and therefore had to be excluded from the analysis. The mean age was [59±17] years, with 33 % being female. The baseline median Acute Physiology and Chronic Health Evaluation (APACHE) II score of the cohort was 23 (IQR: 17–29) and the median Sequential Organ Failure Assessment (SOFA) score was 12 (IQR: 9–15) in the overall cohort and 12 (IQR: 9–14) in the septic shock cohort.

### Treatment modalities and indications of CS therapy

**Figure 1:**
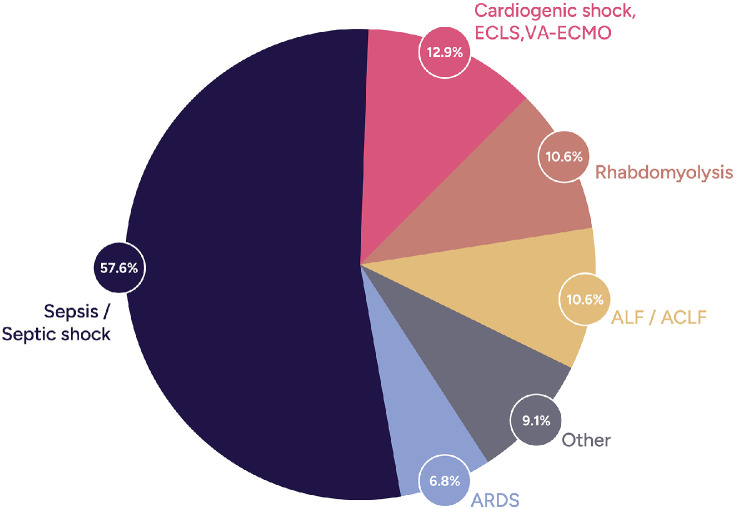
Percentage and type of indications for CytoSorb therapy (multiple indications may apply to one patient). ACLF: Acute-on-chronic liver failure; ALF: Acute liver failure; ARDS: Acute respiratory distress syndrome; ECLS: Extracorporeal life support; ECMO: Extracorporeal membrane oxygenation.

The main indications for CS therapy were septic shock (57.6 %), cardiogenic shock (12.9 %), rhabdomyolysis (10.6 %), acute/acute-on-chronic liver failure (ALF/ACLF, 10.6 %), acute respiratory distress syndrome (ARDS, 6.8 %), and other conditions (9.1 %) with more than one indication for CS in certain patients (Figure 1). Cytokine removal was indicated by far as the most common removal target for hemoadsorption therapy, followed by bilirubin, liver toxins, and myoglobin. The platform used for integration of CS was renal replacement therapy (82.8 %), stand-alone hemoperfusion (9.8 %), and extracorporeal membrane oxygenation (7.4 %). The median number of CS adsorbers used per patient was 2 (IQR:1–3), 39.2 % of patients had a single CS device used, whereas 22.1 % of patients received at least 4 devices or more. The median CS treatment duration was 24 (IQR:18.0–49.8) h.

### Mortality and secondary outcomes

Significant reductions in plasma levels of lactate and creatinine were observed after overall CS treatment. The median lactate level decreased significantly from 2.6 (IQR: 1.7–5.1) mmol/L in the 24 h before CS treatment to 1.5 (IQR: 1.1–2.3) mmol/L in the 24 h post-CS treatment (*P* <0.0001), and creatinine level from 2.29 (IQR: 1.40–3.18) mg/dL to 1.45 (IQR: 0.99–2.17) mg/dL (*P* <0.0001). In the septic shock cohort, lactate levels decreased significantly from a median of 3.3 (IQR: 1.8–6.7) mmol/L to 1.6 (IQR: 1.2–2.9) mmol/L (*P* <0.0001).

Median interleukin-6 (IL-6) decreased significantly from 862.4 (IQR: 142–97,457) pg/mL with a maximum of over 500,000 pg/mL in the 24 h before CS treatment to 202.8 (IQR: 42–3247) pg/mL in the 24 h post-CS treatment (*P* <0.0001). A drop in C-reactive protein (CRP) was observed but did not reach statistical significance (from 13.6 [IQR: 3.0–23.1] mg/dL to 10.74 [IQR: 3.6–17.9] mg/dL, *P*=0.0668).

**Figure 2:**
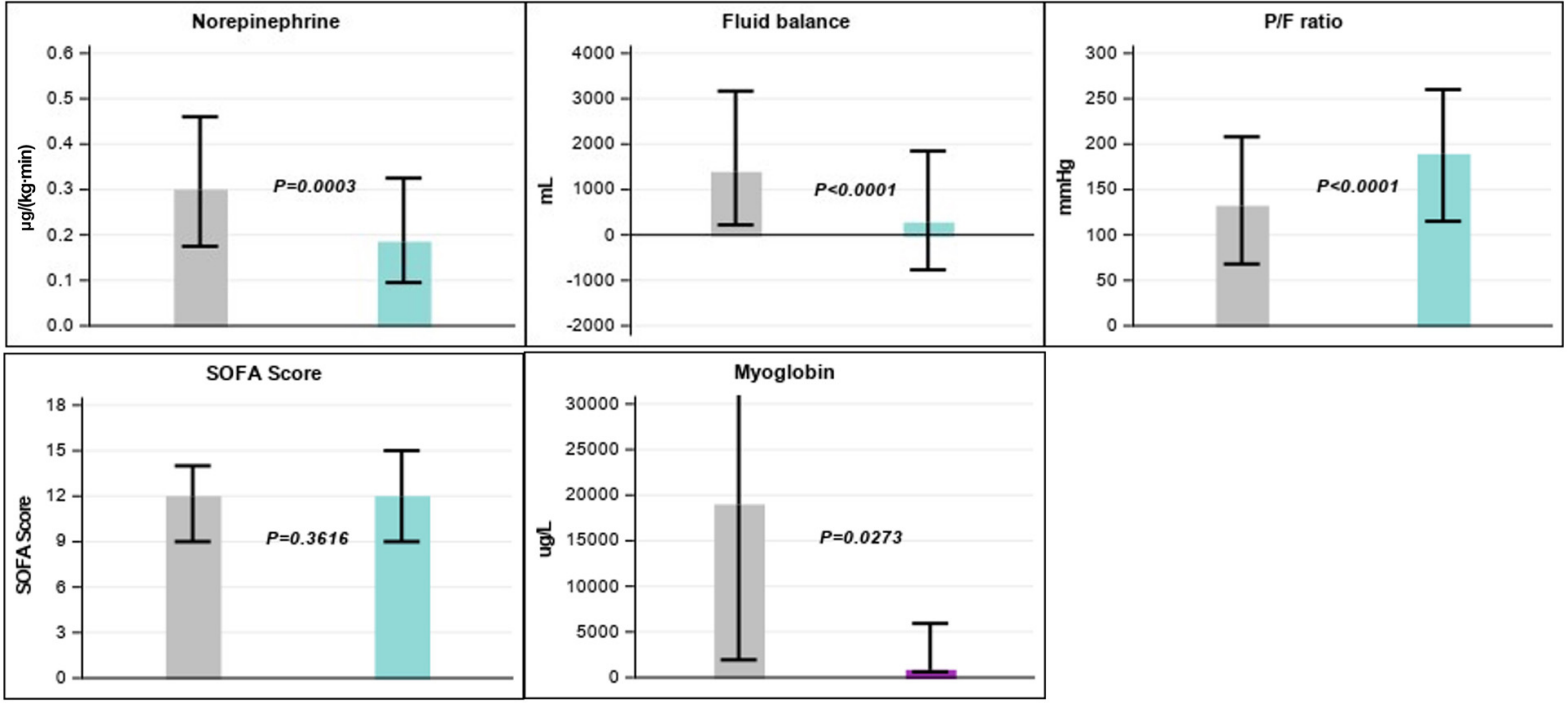
Changes in norepinephrine, fluid balance and P/F ratio, SOFA score and myoglobin in 24 h periods before (gray) *vs.* after CytoSorb^Ⓡ^ treatment (blue). P/F ratio: Ratio of partial pressure of oxygen in arterial blood to the fraction of inspiratory oxygen concentration; SOFA: Sequential organ failure assessment.

The median fluid balance decreased significantly from 1386 mL (IQR: 220–3168) in the 24 h before CS treatment to 275 (IQR: -768–1846) mL in the 24 h post-CS treatment (*P* <0.0001) (Figure 2). In addition, there was a significant reduction in median norepinephrine dosage from 0.30 (IQR: 0.18–0.46) µg/(kg·min) to 0.19 (IQR: 0.10–0.33) µg/(kg·min) (*P*=0.0003). Oxygenation improved significantly over the course of treatment, with the median P/F ratio increasing from 132 (IQR: 68–208) mmHg to 189 (IQR: 115–260) mmHg (*P* <0.0001); however, SOFA score remained unchanged (*P*=0.3616).

In patients with rhabdomyolysis, median myoglobin levels significantly decreased from 18,976 (IQR: 1934–34,275) µg/L to 835 (IQR: 623–5925) µg/L (*P*=0.0273). However, in the liver failure group, the decrease in median bilirubin levels from 7.28 (IQR: 4.2–15.5) mg/dL to 6.11 (IQR: 4.7–8.6) mg/dL did not reach statistical significance (*P*=0.1099), due to the relatively small sample size. The median length of ICU stay for the ICU-survival patients was 20 (IQR: 11–33) days. The ICU mortality rate was 35.0 % in the overall cohort and 37.5 % in the septic shock cohort.

### Safety

Regarding mean duration of treatment per adsorber, there was no negative safety impact regarding loss of platelets or albumin observed with shorter mean duration per adsorber, or longer overall treatment duration, while the drop in platelets was highly impacted by the underlying disease. Median platelet count showed a significant decrease in both the septic shock (122 × 10^9^/L in the 24 h pre-CS treatment *vs.* 52.5 × 10^9^/L in the 24 h post-CS; *P* <0.0001) and liver failure cohorts (110.5 × 10^9^/L in the 24 h pre-CS treatment *vs.* 71.5 × 10^9^/L in the 24 h post-CS; *P*=0.013), whereas no significant change was observed in the rhabdomyolysis cohort (137 × 10^9^/L in the 24 h pre-CS treatment *vs.* 97 × 10^9^/L in the 24 h post-CS; *P*=0.7224). Overall, the median albumin levels did not change significantly during the course of the treatment (*P*=0.5744). No serious adverse device effects or device deficiencies were reported.

## Discussion

In this interim analysis of the COSMOS registry, we examined a diverse cohort of 150 critically ill patients treated with CS therapy across multiple European centers. The indications for CS therapy predominantly included septic shock, cardiogenic shock, rhabdomyolysis, ALF/ACLF, and ARDS. These indications align with the established applications of CS in managing systemic inflammatory responses and multi-organ dysfunction, as supported by previous studies.^[^^19^^]^

Our findings indicate significant reductions in lactate, suggesting improved tissue perfusion and metabolic function, as elevated lactate levels are often indicative of tissue hypoxia and are a marker of poor prognosis in septic shock.^[^^20^^]^ Similarly, a decrease in creatinine levels was observed over the course of treatment. Of course, the exact contribution of CS therapy to these findings is unclear since most patients were also treated with concomitant CRRT. Particularly, the improvement in creatinine is likely to be attributed to removal by RRT. However, published data in rhabdomyolysis patients show enhanced renal function recovery with the use of hemoadsorption, which may partly explain decreasing creatinine levels not only as a primary treatment effect of RRT but also as a potential secondary effect of hemoadsorption.^[^^21^^]^

The findings from the COSMOS registry align closely with previously published data from large-scale CS registry studies while also providing new insights into emerging clinical applications. The significant reduction in IL-6 levels following CS therapy confirms results from the previous registry results where IL-6 reductions were consistently observed across various patient populations, reinforcing the anti-inflammatory potential of CS treatment in critically ill patients.^[^^22^^]^ However, the COSMOS registry reported exceptionally high baseline IL-6 values in some patients (up to >500,000 pg/mL), which exceed typical levels observed in earlier studies.

In addition, the COSMOS data provide new information on the course of physiologic parameters such as the P/F ratio and fluid balance. These variables have not been prominently featured in earlier publications, indicating the development of a deeper understanding of relevant clinical outcome parameters to monitor CS effects over time. Excessive fluid resuscitation can lead to complications such as resuscitation injury, gastrointestinal and cardiac complications, increased extremity compartment pressures, coagulation disturbances, electrolyte imbalance, hypothermia, and abdominal compartment syndrome.^[^^23^^]^ In general, fluid overload is associated with worse outcomes in critically ill patients.^[^^24^^]^ Significant reduction in fluid balance post-CS treatment is particularly relevant in the context of ARDS, where improving oxygenation is a critical therapeutic goal.^[^^25^^]^ Furthermore, fluid accumulation syndrome is also gaining more interest in septic shock patients.^[^^26^^]^ The significant improvement in the P/F ratio is in line with results from the corona-virus infectious disease 2019 ECMO cohort in the CytoSorb Therapy in COVID-19 (CTC) registry and suggests enhanced oxygenation and respiratory function following CS therapy, which may be partly attributed to the improved fluid balance.^[^^27^^]^ The same finding has also been previously published in patients with severe ARDS where CS therapy was shown to contribute to increased P/F ratio.^[^^28^^,^^29^^]^ However, improvement in fluid balance and subsequently lung function would have to be attributed largely to direct volume depletion by CRRT. With increasing patient numbers in the registry, it will be interesting to better examine the impact of CS on these parameters not only in patients without CRRT but also in patients requiring ECMO, since the concept of “enhanced lung rest” with the early initiation of ECMO plus CS has been previously suggested.^[^^27^^,^^30^^]^

Notably, the reduction in fluids was achieved without increasing norepinephrine. Indeed, the opposite was the case, with the decreased need for norepinephrine further supporting hemodynamic stabilization achieved with CS therapy. The reduction in vasopressor dependency is crucial, as vasopressors can lead to sympathetic overstimulation, cardiac dysrhythmias, impaired microcirculatory circulation, motility disturbance of the gut, and interference with the immune system,^[^^31^^]^ and high doses of norepinephrine are linked to adverse outcomes.^[^^32^^]^ Clinical data with CS in septic patients support the idea that reducing inflammatory mediators, along with other yet unknown substances, may lead to less vasodilatation and less capillary leakage,^[^^33^^]^ resulting in improved hemodynamics, decreased need for catecholamines, and reversal of shock.^[^[34], [35], [36]^]^

Comparison of clinical findings in ICU survivors *vs.* non-survivors showed a greater absolute change in norepinephrine in the survivor group. Although CS was terminated at still relatively high norepinephrine levels and only a median of 2 adsorbers have been used, further improvement might have been possible with continued treatment and more frequent adsorber exchanges as shown in other studies.^[^^34^^,^^37^^,^^38^^]^

Moreover, COSMOS captures a wider spectrum of clinical applications, including patient subgroups that were underrepresented in earlier registries, such as individuals with rhabdomyolysis. The significant decrease in myoglobin levels post-treatment highlights the efficacy of CS in removing myoglobin, a large molecule that can cause renal damage if not adequately cleared.^[^^39^^]^ This finding is in line with other studies demonstrating significant myoglobin removal with CS therapy.^[^^10^^,^^40^^]^ However, given the low number of cases with myoglobin measurements (*n* = 9), the results should be interpreted cautiously.

Patients with liver failure were also included in the registry; however, they represent a more homogeneous group compared to previous registry data collections.^[^^41^^]^ Critical liver diseases, such as acute liver failure (ALF) and acute-on-chronic liver failure (ACLF), have high mortality rates^[^^42^^,^^43^^]^ with liver transplantation being the only definitive treatment. Previous studies with CS use have shown effective removal of bilirubin^[^[44], [45], [46]^]^ and bile acids.^[^^47^^,^^48^^]^ Although the decrease in bilirubin levels in the liver failure group of the current analysis did not reach statistical significance, the trend suggests a potential benefit. The median pre-treatment plasma bilirubin level of 7.28 mg/dL may have been too low for substantial removal by the adsorber, given its concentration-dependent characteristics.^[^^49^^]^ Other studies reported much higher initial bilirubin levels,^[^^9^^,^^50^^,^^51^^]^ which likely enabled more effective removal.

The median APACHE II and SOFA scores, which are widely used to predict mortality in ICU patients, were relatively high in the COSMOS cohort, indicating severe illness. The baseline median APACHE II score of 23 corresponds to an expected mortality in nonoperative patients of 40 %.^[^^16^^]^ The median SOFA score of 12 as an initial score corresponds to a mortality of 95.2 % in the general ICU population,^[^^17^^]^ whereas a more recent study specifically focusing on adults with suspected infection predicted an in-hospital mortality of around 50 % for a SOFA of 12.^[^^18^^]^ The ICU mortality rate in the overall cohort was 35 %, and for the sepsis cohort was 37.5 %, both of which are therefore lower than the predicted mortality from the standardized risk scores.^[^[16], [17], [18]^]^ These favorable survival rates are in contrast to a meta-analysis.^[^^12^^]^ The median ICU stay of 20 days reflects the critical nature of these patients, consistent with literature showing prolonged ICU stays in patients with severe sepsis and multi-organ failure.^[^^52^^,^^53^^]^ Given the heterogeneity of the study population, the relevance of SOFA scores may be diminished, so the lack of change in SOFA score does not allow for final conclusions.

The significant drop in platelet count observed in the septic shock and liver failure cohorts could be a concern, as thrombocytopenia is associated with increased mortality in critically ill patients.^[^^54^^]^ However, thrombocytopenia is a known manifestation of septic shock and liver failure, and the drops in platelet count may be to a significant extent related to the underlying illness rather than just the use of the CS device.^[^^55^^,^^56^^]^ Overall, platelet loss has been reported with extracorporeal treatments,^[^^57^^]^ warranting further investigation to balance therapeutic efficacy and potential adverse effects.

### Clinical implications

The findings highlight potential critical considerations for fluid management and hemodynamic support in critically ill patients in the ICU setting. The evidence that excessive fluid resuscitation can lead to multiple complications underscores the need for updated fluid management protocols. Hemoadsorption could be an effective adjunctive therapy in managing fluid overload and enhancing oxygenation in ARDS patients.

The observed reduction in norepinephrine requirements with CS therapy, indicating hemodynamic stabilization, further supports the potential of hemoadsorption therapies in reducing vasopressor dependency. These benefits appear to be most pronounced when CS therapy is initiated early in the course of illness and before irreversible organ failure is established. Therefore, incorporating CS early in ICU treatment protocols could lead to more effective management of septic shock and other conditions requiring vasopressor support.

In summary, our study highlights the potential benefits of CS therapy in managing critically ill patients with severe systemic inflammation and multi-organ involvement. The significant improvements in laboratory and clinical parameters associated with the treatment, along with the favorable survival rates as compared with score-predicted rates, suggest that CS may be an effective adjunctive therapy in critical care.

### Limitations

At the time of this interim analysis, datasets in some patients were not complete, resulting in limited patient numbers for some of the performed analyses. The incomplete documentation in a subset of patients reflects the interim nature of this data analysis, as data entry was still ongoing at the time of extraction from the registry. In addition, the total amount of purified blood as a major determinant of the applied hemoadsorption dose would have been an interesting parameter to examine and will be included only in future analyses of the registry. In addition, the current patient cohort is highly heterogeneous, with varying indications for CS use. As this is real-world data from a registry, no strict inclusion criteria were applied, and no specific cut-offs for inflammatory markers were set, which may have contributed to an even more diverse patient selection. Furthermore, there is no control group in the registry and therefore the reported findings must be considered as preliminary and interpreted with the necessary caution. Further controlled studies, including randomized trials, are needed to expand on these findings.

## Conclusions

The International COSMOS registry offers real-world data encompassing a variety of indications and integration platforms of the CS device. The addition of CS treatment to standard therapy was associated with significant reductions in lactate, creatinine, myoglobin levels, and norepinephrine requirements and led to significant improvements in fluid balance and oxygenation. Observed survival was favorable compared to risk score-based predictions. As the registry continues to evolve, it is anticipated to provide more valuable insights into critical aspects of hemoadsorption therapy, including timing, dosing, and patient selection.

## CRediT authorship contribution statement

**Ricard Ferrer:** Writing – original draft, Supervision, Methodology, Conceptualization. **Matthias Thielmann:** Writing – review & editing. **Andreas Kribben:** Writing – review & editing. **Moritz Unglaube:** Writing – review & editing. **Bartosz Tyczynski:** Writing – review & editing. **Julian Kreutz:** Writing – review & editing. **Andreas Baumann:** Writing – review & editing. **Ulf Guenther:** Writing – review & editing. **Dietrich Henzler:** Writing – review & editing. **Thomas Kirschning:** Writing – review & editing. **Aschraf El-Essawi:** Writing – review & editing. **Thomas Günther:** Writing – review & editing. **Martin Bellgardt:** Writing – review & editing. **Gabriella Bottari:** Writing – review & editing. **Filippo Aucella:** Writing – review & editing. **Jorge Hidalgo:** Writing – review & editing, Supervision. **Jean-Louis Teboul:** Writing – review & editing, Supervision. **Dana Tomescu:** Writing – review & editing. **Teresa Klaus:** Writing – original draft, Visualization, Methodology, Conceptualization. **Weihong Fan:** Visualization, Formal analysis. **Jörg Scheier:** Conceptualization. **Efthymios N. Deliargyris:** Supervision. **Fabio Silvio Taccone:** Supervision, Methodology, Conceptualization.

## References

[1] Hawchar F., Tomescu D., Trager K., Joskowiak D., Kogelmann K., Soukup J. (2022). Hemoadsorption in the critically ill-final results of the International CytoSorb Registry. PLoS One.

[2] Marshall J.C. (2001). Inflammation, coagulopathy, and the pathogenesis of multiple organ dysfunction syndrome. Crit Care Med.

[3] Ronco C., Bagshaw S.M., Bellomo R., Clark W.R., Husain-Syed F., Kellum J.A. (2021). Extracorporeal blood purification and organ support in the critically ill patient during COVID-19 pandemic: expert review and recommendation. Blood Purif.

[4] Ronco C., Tetta C., Mariano F., Wratten M.L., Bonello M., Bordoni V. (2003). Interpreting the mechanisms of continuous renal replacement therapy in sepsis: the peak concentration hypothesis. Artif Organs.

[5] Rimmele T., Kellum J.A. (2011). Clinical review: blood purification for sepsis. Crit Care.

[6] Ronco C., Chawla L., Husain-Syed F., Kellum J.A. (2023). Rationale for sequential extracorporeal therapy (SET) in sepsis. Crit Care.

[7] Mitzner S., Kogelmann K., Ince C., Molnar Z., Ferrer R., Nierhaus A. (2023). Adjunctive hemoadsorption therapy with cytosorb in patients with septic/vasoplegic shock: a best practice consensus statement. J Clin Med.

[8] Jansen A., Waalders N.J.B., van Lier D.P.T., Kox M., Pickkers P. (2023). CytoSorb hemoperfusion markedly attenuates circulating cytokine concentrations during systemic inflammation in humans *in vivo*. Crit Care.

[9] Haselwanter P., Scheiner B., Balcar L., Semmler G., Riedl-Wewalka M., Schmid M. (2024). Use of the CytoSorb adsorber in patients with acute-on-chronic liver failure. Sci Rep.

[10] Scharf C., Liebchen U., Paal M., Irlbeck M., Zoller M., Schroeder I. (2021). Blood purification with a cytokine adsorber for the elimination of myoglobin in critically ill patients with severe rhabdomyolysis. Crit Care.

[11] Hassan K., Geidel S., Zamvar V., Tanaka K., Knezevic-Woods Z., Wendt D. (2023). Intraoperative ticagrelor removal via hemoadsorption during on-pump coronary artery bypass grafting. JTCVS Open.

[12] Becker S., Lang H., Vollmer Barbosa C., Tian Z., Melk A., Schmidt B.M.W (2023). Efficacy of CytoSorb(R): a systematic review and meta-analysis. Crit Care.

[13] Sherman R.E., Anderson S.A., Dal Pan G.J., Gray G.W., Gross T., Hunter N.L. (2016). Real-world evidence – What is it and what can it tell us?. N Engl J Med.

[14] Garrison L.P., Neumann P.J., Erickson P., Marshall D., Mullins C.D (2007). Using real-world data for coverage and payment decisions: the ISPOR Real-World Data Task Force report. Value Health.

[15] International Organization for Standardization (ISO) (2020). ISO 14155:2020 clinical investigation of medical devices for human subjects — Good clinical practice. https://www.iso.org/standard/71690.html.

[16] Sungono V., Hariyanto H., Soesilo T.E.B., Adisasmita A.C., Syarif S., Lukito A.A. (2022). Cohort study of the APACHE II score and mortality for different types of intensive care unit patients. Postgrad Med J.

[17] Palmowski L., Nowak H., Witowski A., Koos B., Wolf A., Weber M. (2024). Assessing SOFA score trajectories in sepsis using machine learning: a pragmatic approach to improve the accuracy of mortality prediction. PLoS One.

[18] Raith E.P., Udy A.A., Bailey M., McGloughlin S., MacIsaac C., Bellomo R. (2017). Prognostic accuracy of the SOFA score, SIRS criteria, and qSOFA score for in-hospital mortality among adults with suspected infection admitted to the intensive care unit. JAMA.

[19] Schadler D., Pausch C., Heise D., Meier-Hellmann A., Brederlau J., Weiler N. (2017). The effect of a novel extracorporeal cytokine hemoadsorption device on IL-6 elimination in septic patients: a randomized controlled trial. PLoS One.

[20] Jansen T.C., van Bommel J., Bakker J. (2009). Blood lactate monitoring in critically ill patients: a systematic health technology assessment. Crit Care Med.

[21] Grafe C., Liebchen U., Greimel A., Maciuga N., Bruegel M., Irlbeck M. (2023). The effect of cytosorb(R) application on kidney recovery in critically ill patients with severe rhabdomyolysis: a propensity score matching analysis. Ren Fail.

[22] Hawchar F., Laszlo I., Oveges N., Trasy D., Ondrik Z., Molnar Z. (2019). Extracorporeal cytokine adsorption in septic shock: a proof of concept randomized, controlled pilot study. J Crit Care.

[23] Cherkas D. (2011). Traumatic hemorrhagic shock: advances in fluid management. Emerg Med Pract.

[24] Boyd J.H., Forbes J., Nakada T.A., Walley K.R., Russell J.A. (2011). Fluid resuscitation in septic shock: a positive fluid balance and elevated central venous pressure are associated with increased mortality. Crit Care Med.

[25] Fan E., Del Sorbo L., Goligher E.C., Hodgson C.L., Munshi L., Walkey A.J. (2017). An Official American Thoracic Society/European Society of Intensive Care Medicine/Society of Critical Care Medicine Clinical Practice guideline: mechanical ventilation in adult patients with acute respiratory distress syndrome. Am J Respir Crit Care Med.

[26] Pfortmueller C.A., Dabrowski W., Wise R., van Regenmortel N., Malbrain M. (2024). Fluid accumulation syndrome in sepsis and septic shock: pathophysiology, relevance and treatment-a comprehensive review. Ann Intensive Care.

[27] Hayanga J.W.A., Song T., Durham L., Garrison L., Smith D., Molnar Z. (2023). Extracorporeal hemoadsorption in critically ill COVID-19 patients on VV ECMO: the CytoSorb therapy in COVID-19 (CTC) registry. Crit Care.

[28] Akil A., Napp L.C., Rao C., Klaus T., Scheier J., Pappalardo F. (2022). Use of CytoSorb(c) hemoadsorption in patients on veno-venous ECMO support for severe acute respiratory distress syndrome: a systematic review. J Clin Med.

[29] Tomescu D., Popescu M., Akil A., Nassiri A.A., Wunderlich-Sperl F., Kogelmann K. (2023). The potential role of extracorporeal cytokine removal with CytoSorb(R) as an adjuvant therapy in acute respiratory distress syndrome. Int J Artif Organs.

[30] Song Z., Tu D., Tang G., Liu N., Tai Z., Yang J. (2023). Hemophagocytic lymphohistiocytosis and disseminated intravascular coagulation are underestimated, but fatal adverse events in chimeric antigen receptor T-cell therapy. Haematologica.

[31] Bangash M.N., Kong M.L., Pearse R.M. (2012). Use of inotropes and vasopressor agents in critically ill patients. Br J Pharmacol.

[32] Domizi R., Calcinaro S., Harris S., Beilstein C., Boerma C., Chiche J.D. (2020). Relationship between norepinephrine dose, tachycardia and outcome in septic shock: a multicentre evaluation. J Crit Care.

[33] David S., Thamm K., Schmidt B.M., Falk C.S., Kielstein J.T. (2017). Effect of extracorporeal cytokine removal on vascular barrier function in a septic shock patient. J Intensive Care.

[34] Friesecke S., Stecher S.S., Gross S., Felix S.B., Nierhaus A. (2017). Extracorporeal cytokine elimination as rescue therapy in refractory septic shock: a prospective single-center study. J Artif Organs.

[35] Kogelmann K., Jarczak D., Scheller M., Druner M. (2017). Hemoadsorption by CytoSorb in septic patients: a case series. Crit Care.

[36] Hawchar F., Rao C., Akil A., Mehta Y., Rugg C., Scheier J. (2021). The potential role of extracorporeal cytokine removal in hemodynamic stabilization in hyperinflammatory shock. Biomedicines.

[37] Rugg C., Klose R., Hornung R., Innerhofer N., Bachler M., Schmid S. (2020). Hemoadsorption with CytoSorb in septic shock reduces catecholamine requirements and in-hospital mortality: a single-center retrospective 'genetic' matched analysis. Biomedicines.

[38] Mariano F., Greco D., Depetris N., Mella A., Sciarrillo A., Stella M. (2024). CytoSorb(R) in burn patients with septic shock and acute kidney injury on continuous kidney replacement therapy is associated with improved clinical outcome and survival. Burns.

[39] Zimmerman J.L., Shen M.C. (2013). Rhabdomyolysis. Chest.

[40] Albrecht F., Schunk S., Fuchs M., Volk T., Geisel J., Fliser D. (2024). Rapid and effective elimination of myoglobin with CytoSorb(R) hemoadsorber in patients with severe rhabdomyolysis. Blood Purif.

[41] Ocskay K., Tomescu D., Faltlhauser A., Jacob D., Friesecke S., Malbrain M. (2021). Hemoadsorption in 'liver indication'-analysis of 109 patients' data from the CytoSorb international registry. J Clin Med.

[42] Garcia Martinez J.J., Bendjelid K. (2018). Artificial liver support systems: what is new over the last decade?. Ann Intensive Care.

[43] Singh H., Pai C.G. (2015). Defining acute-on-chronic liver failure: east, West or Middle ground?. World J Hepatol.

[44] Riva I., Marino A., Valetti T.M., Marchesi G., Fabretti F. (2024). Extracorporeal liver support techniques: a comparison. J Artif Organs.

[45] Scharf C., Liebchen U., Paal M., Becker-Pennrich A., Irlbeck M., Zoller M. (2021). Successful elimination of bilirubin in critically ill patients with acute liver dysfunction using a cytokine adsorber and albumin dialysis: a pilot study. Sci Rep.

[46] Tomescu D., Popescu M., David C., Sima R., Dima S. (2021). Haemoadsorption by CytoSorb(R) in patients with acute liver failure: a case series. Int J Artif Organs.

[47] Hartmann J., Harm S. (2018). Removal of bile acids by extracorporeal therapies: an *in vitro* study. Int J Artif Organs.

[48] Dhokia V.D., Madhavan D., Austin A., Morris C.G (2019). Novel use of Cytosorb haemadsorption to provide biochemical control in liver impairment. J Intensive Care Soc.

[49] Song M., Winchester J., Albright R.L., Capponi V.J., Choquette M.D., Kellum J.A. (2004). Cytokine removal with a novel adsorbent polymer. Blood Purif.

[50] Popescu M., David C., Marcu A., Olita M.R., Mihaila M., Tomescu D. (2023). Artificial liver support with CytoSorb and MARS in liver failure: a retrospective propensity matched analysis. J Clin Med.

[51] Sekandarzad A., Graf E., Prager E.P., Luxenburger H., Staudacher D.L., Wengenmayer T. (2024). Cytokine adsorption in patients with acute-on-chronic liver failure (CYTOHEP): a single center, open-label, three-arm, randomized, controlled intervention pilot trial. Artif Organs.

[52] Vincent J.L., Moreno R., Takala J., Willatts S., De Mendonca A., Bruining H. (1996). The SOFA (sepsis-related organ failure assessment) score to describe organ dysfunction/failure. On behalf of the working group on sepsis-related problems of the European Society of Intensive Care Medicine. Intensive Care Med.

[53] Knaus W.A., Draper E.A., Wagner D.P., Zimmerman J.E. (1985). APACHE II: a severity of disease classification system. Crit Care Med.

[54] Vanderschueren S., De Weerdt A., Malbrain M., Vankersschaever D., Frans E., Wilmer A. (2000). Thrombocytopenia and prognosis in intensive care. Crit Care Med.

[55] Cheng J., Zeng H., Chen H., Fan L., Xu C., Huang H. (2023). Current knowledge of thrombocytopenia in sepsis and COVID-19. Front Immunol.

[56] Ye Q., Wang X., Xu X., Chen J., Christiani D.C., Chen F. (2024). Serial platelet count as a dynamic prediction marker of hospital mortality among septic patients. Burns Trauma.

[57] Raasveld S.J., van den Oord C., Schenk J., van den Bergh W.M., Oude Lansink-Hartgring A., van der Velde F. (2023). The interaction of thrombocytopenia, hemorrhage, and platelet transfusion in venoarterial extracorporeal membrane oxygenation: a multicenter observational study. Crit Care.

